# Supplementary material for: Local synthesis of dynein cofactors matches retrograde transport to acutely changing demands
Source: Nat Commun. 2016 Dec 21;7:13865. doi: 10.1038/ncomms13865 (PMC5187584; doi:10.1038/ncomms13865)
Supplement: Supplementary Information — Supplementary Figures. [file ncomms13865-s1.pdf]

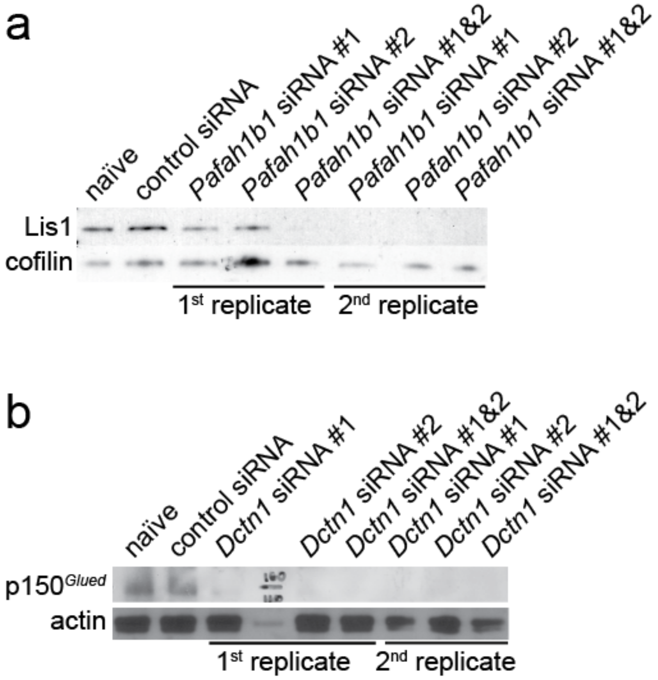

Supplementary Figure 1. Validation of siRNAs
C6 cells were transfected with each two siRNAs targeting *Pafah1b1* (A), *Dctn1* (B) and a non-targeting control siRNA for 72 hours before immunoblot analysis.

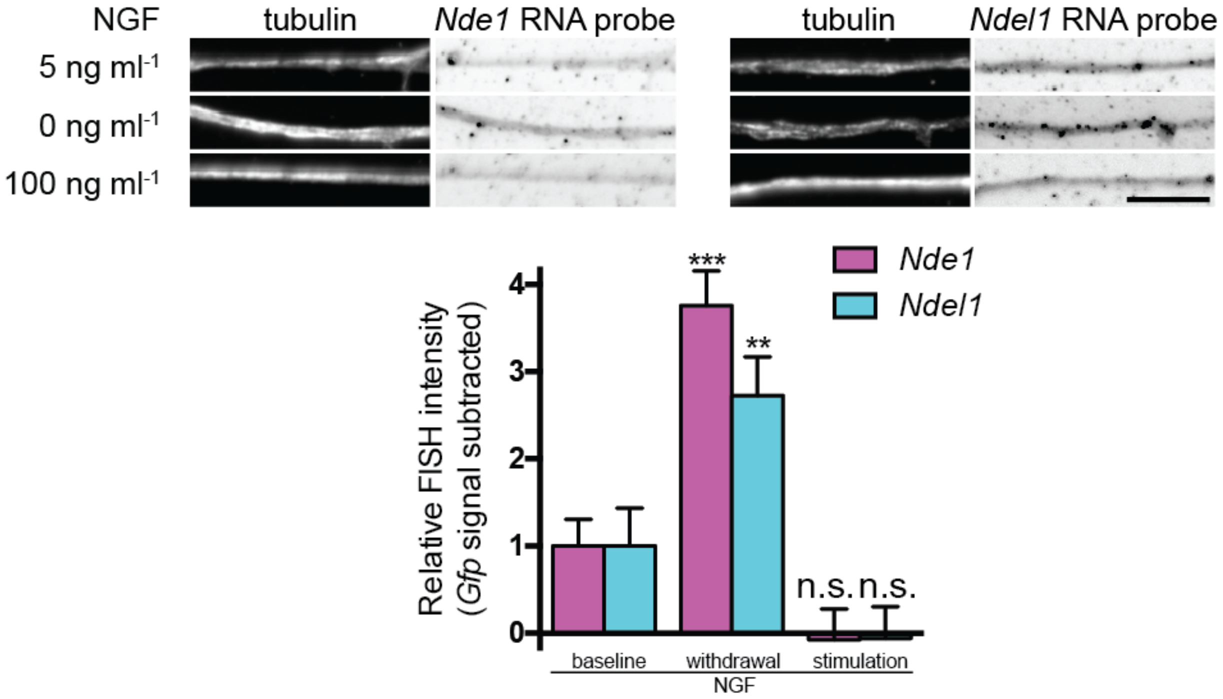

Supplementary Figure 2. FISH for NudE and NudEL transcripts in axons
DRG neurons were cultured in microfluidic chambers for 3 DIV, at which time the NGF concentration in the axonal chamber was changed to 5 ng ml^{-1}. 24 h later, the NGF concentration in the axonal chamber was adjusted to 0, 5, or 100 ng ml^{-1} NGF for 12 h, and axonal *Nde1* or *Ndel1* mRNA levels were determined by FISH. Means ± SEM of 45-60 optical fields per condition. **, p≤0.01; ***, p≤0.001. One-way ANOVA.
Scale bar, 10 µm.

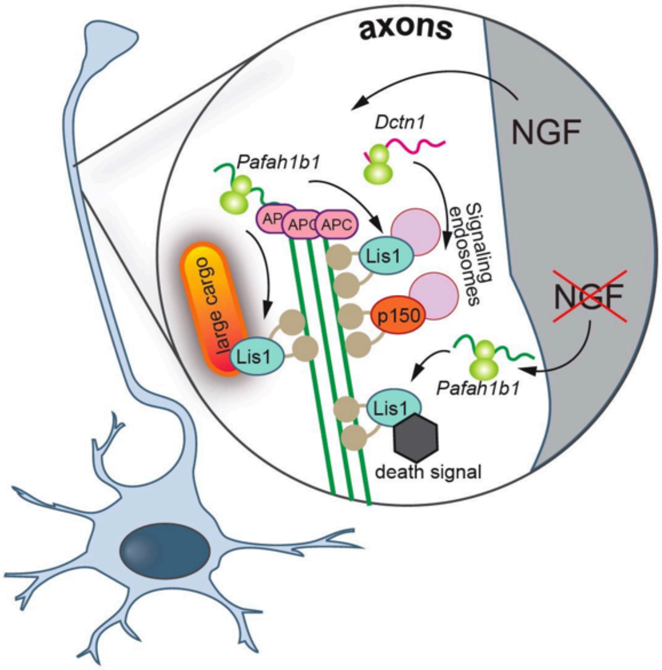

Supplementary Figure 3. Model for the NGF-dependent regulation of retrograde axonal transport through locally produced dynein cofactors

NGF stimulation leads to the axonal translation of *Dctn1* and APC-bound *Pafah1b1*. Locally synthesized Lis1 required for the retrograde transport of vesicular cargoes greater than 1 µm in diameter, and both axonally-derived Lis1 and p150^{Glued} are necessary for the retrograde transport of NGF-signaling endosomes. NGF withdrawal has no effect on p150^{Glued} synthesis but causes the production of Lis1 off not APC-bound Pafah1b1 transcripts. Lis1 produced in response to NGF withdrawal is required for the retrograde transport of a death signal.

a

M 1 2 3 4 5 6 7 8

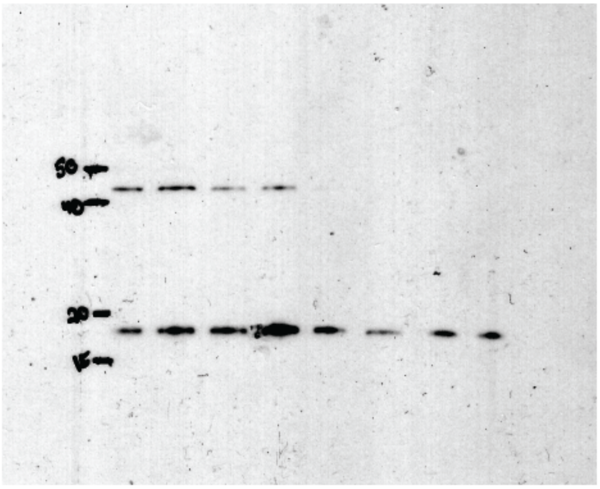

1: naïve
2: control siRNA
3: *Pafah1b1* siRNA #1
4: *Pafah1b1* siRNA #2
5: *Pafah1b1* siRNA #1&2
6: *Pafah1b1* siRNA #1
7: *Pafah1b1* siRNA #2
8: *Pafah1b1* siRNA #1&2

WB: Lis1 and cofilin

b

1 2 3 M 4 5 6 7 8

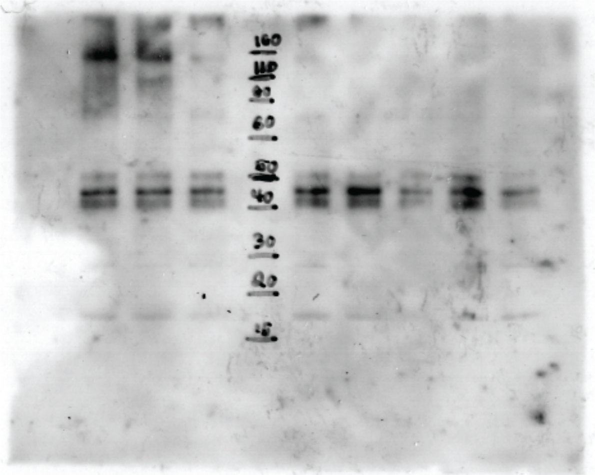

1: naïve
2: control siRNA
3: *Dctn1* siRNA #1
4: *Dctn1* siRNA #2
5: *Dctn1* siRNA #1&2
6: *Dctn1* siRNA #1
7: *Dctn1* siRNA #2
8: *Dctn1* siRNA #1&2

WB: p150^{Glued}

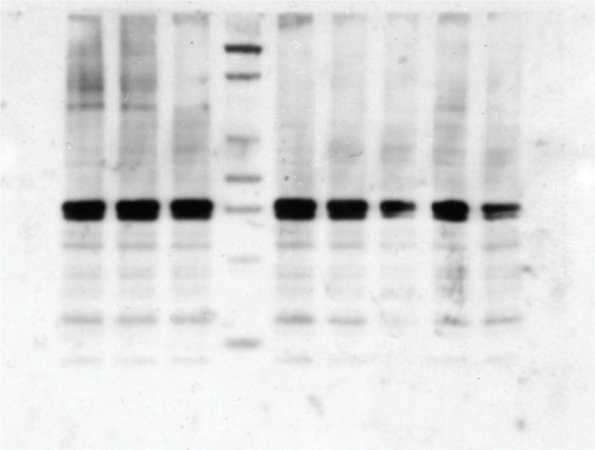

WB: actin

Supplementary Figure 4. Full size images of Western Blots
Full size scans of immunoblots presented in supplementary Fig. 1.
